# Influence of Macrophages on Vascular Invasion of Inflammatory Breast Cancer Emboli Measured Using an In Vitro Microfluidic Multi-Cellular Platform

**DOI:** 10.3390/cancers15194883

**Published:** 2023-10-08

**Authors:** Manasa Gadde, Melika Mehrabi-Dehdezi, Bisrat G. Debeb, Wendy A. Woodward, Marissa Nichole Rylander

**Affiliations:** 1 Walker Department of Mechanical Engineering, The University of Texas at Austin, Austin, TX 78712, USA; manasa_gadde@yahoo.com (M.G.); melikamehrabi@utexas.edu (M.M.-D.); 2 Department of Breast Medical Oncology, The University of Texas MD Anderson Cancer Center, Houston, TX 77030, USA; bgdebeb@mdanderson.org; 3 MD Anderson Morgan Welch Inflammatory Breast Cancer Clinic and Research Program, The University of Texas MD Anderson Cancer Center, Houston, TX 77030, USA; wwoodward@mdanderson.org; 4 Department of Radiation Oncology, The University of Texas MD Anderson Cancer Center, Houston, TX 77030, USA; 5 Department of Biomedical Engineering, The University of Texas at Austin, Austin, TX 78712, USA; 6 Oden Institute for Computational and Engineering Sciences, The University of Texas at Austin, Austin, TX 78712, USA

**Keywords:** inflammatory breast cancer, tumor-associated macrophages, MDA-IBC3, intravasation, vasculature, angiogenesis, lymphangiogenesis, extravasation

## Abstract

**Simple Summary:**

Macrophages, specifically tumor-associated macrophages (TAMs), play a vital role in inflammatory breast cancer (IBC) progression, including cell growth, angiogenesis, and resistance to treatment. Current in vitro models for studying IBC have limitations and do not fully capture the dynamic nature of interactions between macrophages, the tumor, and the microenvironment in IBC. Therefore, we have developed a 3D in vitro model of IBC that incorporates a collagen matrix, functional blood vessels, and THP1 M0, M1, or M2 macrophages, allowing for the study of macrophage-tumor interactions and angiogenesis. Using this platform, it was found that Incorporating TAMs increases the number of new vessel sprouts, permeability of the vessel, matrix porosity, and intravasation of MDA-IBC3 cells. Additionally, IL8 and MMP-9, which are known to promote angiogenesis and tumor invasion, were found to be preferentially secreted in M0 and M2 co-culture platforms.

**Abstract:**

Inflammatory breast cancer (IBC) is an aggressive disease with a poor prognosis and a lack of effective treatments. It is widely established that understanding the interactions between tumor-associated macrophages (TAMs) and the tumor microenvironment is essential for identifying distinct targeting markers that help with prognosis and subsequent development of effective treatments. In this study, we present a 3D in vitro microfluidic IBC platform consisting of THP1 M0, M1, or M2 macrophages, IBC cells, and endothelial cells. The platform comprises a collagen matrix that includes an endothelialized vessel, creating a physiologically relevant environment for cellular interactions. Through the utilization of this platform, it was discovered that the inclusion of tumor-associated macrophages (TAMs) led to an increase in the formation of new blood vessel sprouts and enhanced permeability of the endothelium, regardless of the macrophage phenotype. Interestingly, the platforms containing THP-1 M1 or M2 macrophages exhibited significantly greater porosity in the collagen extracellular matrix (ECM) compared to the platforms containing THP-1 M0 and the MDA-IBC3 cells alone. Cytokine analysis revealed that IL-8 and MMP9 showed selective increases when macrophages were cultured in the platforms. Notably, intravasation of tumor cells into the vessels was observed exclusively in the platform containing MDA-IBC3 and M0 macrophages.

## 1. Introduction

Inflammatory breast cancer (IBC) is a rare and lethal subset of breast cancer characterized using rapid onset and clinical breast skin changes, including redness and edema. The poor prognosis of this disease is linked to the late stage at which inflammatory breast cancer (IBC) is typically detected. These factors, combined with the absence of treatments specifically designed for IBC, contribute to the poor prognosis associated with the disease [1,2,3].

Currently, there is a lack of histological differentiations or biomarkers that can effectively distinguish between IBC tumors and non-IBC tumors with a high level of sensitivity and specificity. However, clinical observations of IBC pathologic specimens indicate that they exhibit higher levels of angiogenesis and metastasis, along with increased involvement of lymph nodes compared to non-IBC cases [4] and extensive lymphovascular emboli contributing to the characteristic breast and skin swelling [5]. Recent studies of the tumor microenvironment and breast stroma demonstrate a role for macrophages in IBC presentation and progression [6,7,8].

Macrophages associated explicitly with tumors, called tumor-associated macrophages (TAMs), have been well studied in breast cancer in general and play a crucial role in the progression of the disease. Their contributions encompass various aspects such as cell proliferation, angiogenesis, metastasis, and even chemoresistance [9,10,11,12,13,14,15,16]. In breast tumors, TAMs have been found to constitute more than 50% of the tumor mass. Numerous clinical studies have established a correlation between a high density of TAMs and poor prognosis in breast cancer cases [17,18,19,20,21]. Recent studies have highlighted the importance of TAMs in the prognosis and progression of IBC. Several studies have demonstrated that high levels of M2 macrophages are associated with poor prognosis and disease progression in inflammatory breast cancer. They found that M2 macrophages promote the development of an immunosuppressive and pro-tumorigenic microenvironment in inflammatory breast cancer, which can lead to more aggressive disease behavior [22,23,24]. Examinations conducted on patient IBC tumors have unveiled a significant buildup of macrophages in the breast tissue surrounding IBC tumors. Furthermore, there is a notable infiltration of tumor-associated macrophages (TAMs) in IBC models where the skin is involved [25]. In a study, Allen et al. cultured both IBC and non-IBC cells in macrophage-conditioned media from U937 macrophages and saw increased migration of cancer cells in the IBC groups compared to non-IBC [9]. An alternative study conducted by another research group revealed that the IBC cell line, SUM149, exhibits structural changes from emboli-like structures to branched-like structures, which induce migration when exposed to media conditioned using CD14+ cells isolated from IBC patients compared to Ham’s F12 and 5% FBS culture media [6]. Subsequent investigations have demonstrated a correlation within IBC between the presence of macrophages, lymph node invasion, and the expression of matrix-degrading proteases, as well as multiple angiogenic and chemotactic factors. [26,27,28]. In particular, one study found that TAMs in IBC tumors express high levels of CCL18, a chemokine known to promote angiogenesis, which was associated with lymph node involvement and poor survival outcomes in IBC patients [29]. These studies demonstrate that macrophages and TAMs play a crucial role in the progression of IBC.

Despite the growing body of research on stromal macrophages and TAMs in IBC, there remains a gap in our knowledge regarding the dynamic nature of these interactions with tumors and the microenvironment. To the best of our knowledge, there are currently no high-throughput methods for studying these interactions in a manner that is both readily modifiable and comprehensive. Typical in vitro models for IBC involve growing tumor spheroids or culturing IBC cells on an extracellular matrix (ECM) layer made of Matrigel or collagen. Despite advancements in creating more intricate IBC models, including co-cultures, these models have not yet accounted for environmental factors like flow, ECM composition, and physiologically relevant geometries [6,9,30,31,32,33,34,35]. There are yet no genetically engineered mouse models that replicate the IBC phenotype. Xenograft IBC models lack the full spectrum of human immune cells and can be expensive and difficult to employ for detailed growth pattern analysis, while 2D culture systems lack the 3D architecture and cellular interactions present in vivo, leading to inaccurate assessments of tumor response to therapies [36]. Elucidation of the dynamic interactions of IBC and macrophages and their role in the progression of IBC could identify viable targets for future IBC therapeutics. Therefore, there is a critical need for experimental systems that will enable the determination of the spatial and temporal dynamics of macrophage interactions with the IBC tumor microenvironment and their role in tumor response and progression. Recognizing vascular invasion by IBC tumor emboli is an integral component of IBC progression; the study of these interactions, inclusive of the vasculature, is paramount.

In this study, we present a 3D in vitro vascularized microfluidic IBC platform to, for the first time, comprehensively capture macrophage dynamics in the context of IBC in a collagen matrix and resulting angiogenesis. This model encompasses multiple aspects of IBC pathology, incorporating a collagen I matrix with a functional, endothelialized vessel, allowing for cellular interactions between THP-1 Macrophages, IBC cells, and the vasculature in a physiologically relevant environment. To validate this platform’s utility and physiologic relevance, we reproduced the angiogenic influence of TAMs on baseline IBC cell induction of vessel sprouting and the development of the clinical hallmark of vascular invasion by IBC emboli [37]. We further report the presence of the macrophages resulted in significant endothelial vessel leakage and a more porous collagen matrix, promoting the intravasation of MDA-IBC3 cells, highlighting the importance of TAM in the progression of IBC.

## 2. Materials and Methods

### 2.1. Cell Culture

For this study, human inflammatory breast cancer (IBC) cell lines MDA-IBC3 and SUM149, as well as the THP-1 monocyte-derived macrophage cell line and telomerase-immortalized human microvascular endothelial (TIME) cells, were employed. To facilitate real-time imaging studies, TIME cells were genetically modified using a lentiviral vector system to express the mCherry red fluorescent protein (kindly provided by Dr. Shay Soker at the Wake Forest Institute for Regenerative Medicine, Winston-Salem, NC). The MDA-IBC3 and SUM149 IBC cell lines, labeled with a green fluorescent protein (GFP), were generously provided by Dr. Wendy Woodward from MD Anderson Cancer Center (Houston, TX, USA). MDA-IBC3 and SUM149 cells were cultured in Ham’s F-12 media supplemented with 10% FBS, 1% antibiotic-antimycotic, 1 µg/mL hydrocortisone, and 5 µg/mL insulin. TIME cells were cultured in endothelial cell growth medium 2 (EGM2, Promocell). The M0, M1, and M2 THP-1 macrophages were obtained from ATCC (Manassas, VA, USA). Briefly, THP-1 human monocyte cells (ATCC, Manassas, VA; TIB-202) were cultured in Roswell Park Memorial Institute (RPMI) 1640 medium (Corning Cellgro, Manassas, VA, USA), supplemented with 10% fetal bovine serum (FBS), 1% sodium pyruvate, and 1% antibiotic-antimycotic. The differentiation process to M0-THP1 macrophages involved a 24 h incubation of THP-1 monocytes with 320 nM phorbol 12-myristate 13-acetate (PMA, Sigma, St. Louis, MO, USA, P8139). For the polarization of macrophages into the M1 phenotype, they were exposed to 20 ng/mL of IFN-γ (R&D Systems, Minneapolis, MN, USA, #285-IF) and 10 pg/mL of LPS (Sigma, #8630). On the other hand, M2 polarization of macrophages was achieved using incubation with 20 ng/mL of interleukin 4 (R&D Systems, #204-IL) and 20 ng/mL of interleukin 13 (R&D Systems, #213-ILB). All cell cultures used in this study were maintained in a 5% CO_2_ atmosphere at 37 °C in an incubator [38].

### 2.2. In Vitro 3D Tumor Platform Fabrication

The in vitro 3D tumor microfluidic platforms employed in this study consisted of a rat tail collagen type I matrix. These platforms were seeded with either MDA-IBC3 or SUM149 cells, as well as THP-1-derived macrophages conditioned to M0, M1, and M2 phenotypes, following the procedure described earlier. Additionally, a hollow vessel lined with mCherry-labeled TIME cells was incorporated into a polydimethylsiloxane (PDMS) scaffold. To prepare the collagen type I matrix, collagen extracted from rat tails was processed according to established protocols [39]. This yielded a stock collagen concentration of 14 mg/mL, which was subsequently neutralized with 10X DMEM, 1N NaOH, and 1X DMEM to achieve a final collagen concentration of 7 mg/mL. This concentration was chosen to closely resemble the reported stiffness of breast tumors in clinical settings [40,41,42,43]. To initiate the experiment, GFP-labeled IBC cells and unlabeled THP-1 cells conditioned in M0, M1, or M2 phenotypes were combined and seeded at a density of 1 × 10^6^ cells/mL each. This cell mixture was then introduced into the neutralized collagen solution with a concentration of 7 mg/mL. The mixture was polymerized around a 22G needle at 37 °C for 25 min. Once the polymerization process was complete, the needle was carefully removed, creating a hollow void. To form the endothelialized vessel lumen, a solution containing 2 × 10^5^ mCherry-labeled TIME cells was injected into the void [40,43,44,45]. In summary, the flow was carefully perfused to expose the endothelium to a wall shear stress (WSS) of 0.01 dyn/cm^2^ for a duration of 36 h. Subsequently, there was a gradual increment in the WSS to 0.1 dyn/cm^2^ for the subsequent 36 h. Once the 72 h graded flow protocol was successfully completed, the in vitro vascularized platforms were subjected to a flow rate of 1 dyn/cm^2^ for a period of 6 h.

### 2.3. Quantification of Endothelial Morphology, Sprouting, and Permeability

Following the 78 h flow treatment, confocal microscopy was employed to capture images of the central plane of the in vitro platforms containing the vessel. To quantify the extent of vessel sprouting in each group, ImageJ analysis plugins called “tubeness” and “skeleton” were utilized. For each group, three independent replicates (n = 3) were analyzed, and the sprouting was measured as sprouting per unit length of the vessel. The statistical significance of the data was determined using a one-way ANOVA test.

Upon completion of the 78 h flow protocol, the analysis of endothelial cell–cell junctions was conducted by performing immunofluorescent staining for platelet endothelial cell adhesion molecule-1 (PECAM-1). PECAM-1, labeled in green, is known to be expressed at endothelial intercellular junctions and plays a crucial role in maintaining endothelial barrier functions [20]. For the staining process, the platforms underwent perfusion with a solution containing 4% paraformaldehyde to ensure fixation of the cells and 0.5% triton-X to enable permeabilization of the cell membranes. Subsequently, the platforms were subjected to an incubation step in a solution consisting of 5% BSA to block any non-specific binding. This was followed by an overnight incubation, during which the platforms were exposed to antibodies specifically targeting PECAM-1, conjugated with Alexa 488 (Abcam, Cambridge, UK, ab215911).

The evaluation of endothelial vessel permeability, influenced by paracrine signaling interactions involving the tumor, macrophages, and vasculature, was conducted by perfusing the channels with serum-free media containing GFP-labeled dextran with a molecular weight of 70 kDa (10 μg/mL) [44,46]. Confocal images were captured at intervals of three minutes, allowing for the measurement of average dextran fluorescence intensity. This intensity measurement was utilized to determine the diffusion permeability coefficient, Pd, following previously established methods [44]. Each platform condition was evaluated using three independent replicates (n = 3), and the resulting permeability factor was expressed as the mean value ± standard deviation. Statistical significance was assessed using a one-way ANOVA test.

### 2.4. Matrix Porosity

The endothelial adhesion to the collagen matrix and its porosity were measured by employing Scanning electron microscopy (SEM). After exposure to the graded flow protocol, the platforms were fixed in an aldehyde mixture overnight at room temperature, followed by fixation with osmium on ice for 4 h. After fixing the platforms, they underwent a dehydration process using a sequence of ethanol solutions (50%, 70%, 95%, and 100%) and then critical point dried by CO_2_. Platforms were coated with a thin layer of platinum-palladium, and SEM imaging was performed with Zeiss Supra40 SEM-Electron Microscope. Porosity was determined by analyzing SEM images of the collagen network using FIJI, an image processing software. Images of the collagen matrix were acquired at 50,000× magnification, and FIJI was used to measure porosity, or the ratio of signal from the collagen fibers to the total imaged area.

### 2.5. Cytokine Analyses

Cytokine analyses were conducted to measure the levels of CD31, ANG1, ANG2, TGF-α, bFGF, PDGF-bb, VEGF-A, EGF, VEGFR3, VEGF-C, TNF-α, IL-8, IL-6, IL-6 Rα, MMP9, MMP2, MMP13. These analyses were performed using a custom human magnetic Luminex assay (R&D Systems) on the effluent from platform perfusion, following the instructions provided by the manufacturer. The obtained data were subjected to statistical analysis using one-way ANOVA, and a 95% confidence criterion was used to determine the significance of the results.

## 3. Results

### 3.1. Angiogenic Sprouting of the Vascular Endothelium

**Figure 1:**
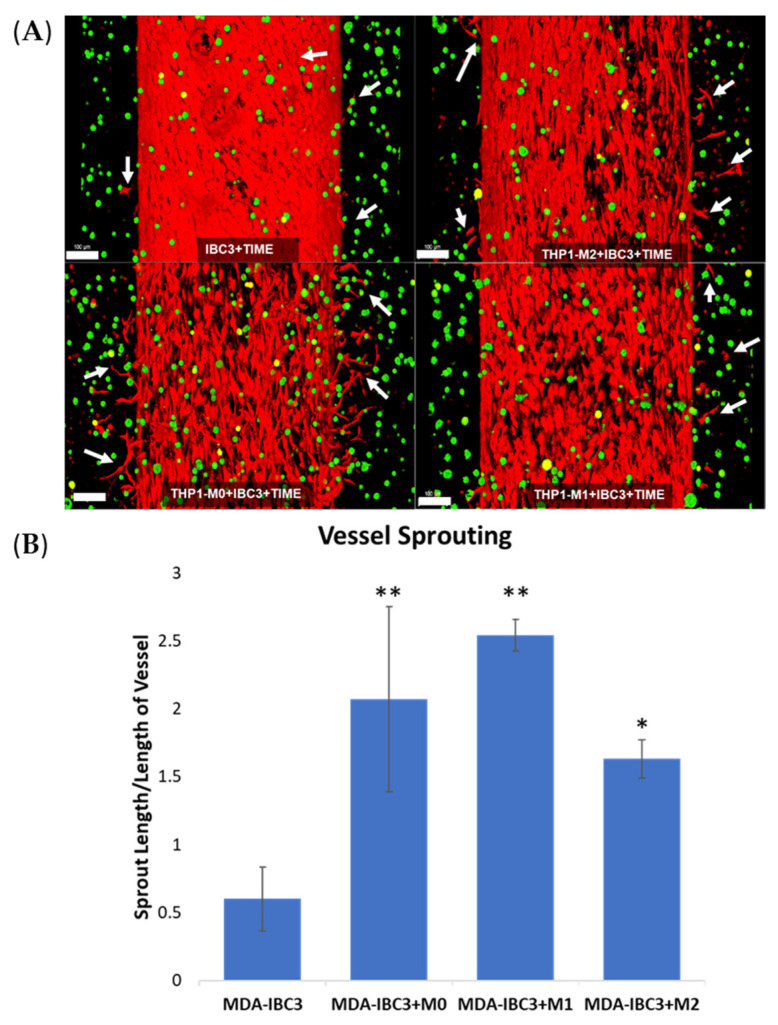
Confocal images of in vitro vascularized breast tumor platforms consisting of MDA-IBC3 cells (green) and within a collagen matrix surrounding a vessel seeded with TIME cells (red). These platforms incorporate the presence or absence of M0, M1, or M2 THP-1 cells. Arrows highlight newly formed vessel sprouts; scale bar: 100 µm (**A**), and quantification of vessel sprouting is represented as sprout length/length of vessel (**B**), * *p* < 0.05, ** *p* < 0.01.

We previously demonstrated that MDA-IBC3 cells promote vessel sprouting compared to non-IBC MDA-231 cells (Gadde et al.). Figure 1 demonstrates that the in vitro vascular MDA-IBC3 breast tumor platforms exhibited an augmentation in vessel sprouting in the presence of macrophages. Figure 1A depicts the endothelial vessels and IBC3 cells with and without the inclusion of M0, M1, and M2 THP-1-derived macrophages, respectively. To quantify the sprouting, Figure 1B employed tubeness and skeleton plugins in ImageJ on confocal-acquired images of the endothelium. This analysis aimed to identify differences in vessel sprouting across various conditions. The results indicated a significant increase in sprouting in platforms with macrophages (M0 *p* < 0.01, M1 *p* < 0.01, M2 *p* < 0.05), regardless of their phenotype, compared to the control platform of MDA-IBC3.

### 3.2. Intravasation of Tumor Cells

**Figure 2:**
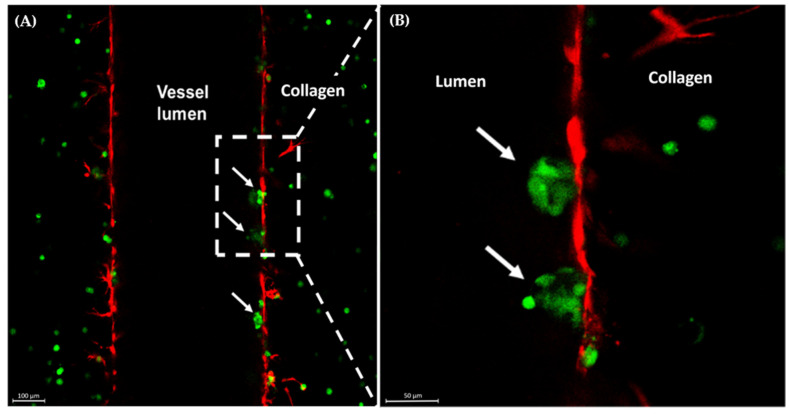
Confocal images of MDA-IBC3+M0 in vitro IBC platforms. Arrows indicate MDA-IBC3 cells (green) crossing the endothelium (red) (**A**); magnified view of the area in the outlined box (**B**).

The swollen, red breast characteristic of IBC presentation is attributed to the invasion of IBC cells as clusters of tumor cells, emboli, into the blood and lymphatic vessels in the breast and skin. Previous research has highlighted the importance of macrophages in tumor cell invasion and has specifically highlighted IL-8 as a key cytokine in tumor cell-induced angiogenesis and invasion [16,47,48]. With the addition of macrophages in the matrix of the MDA-IBC3 platform, we were able to capture physiologic intravasation of emboli into the vessels with cell adhesion to the endothelial layer in the presence of physiologic flow (Figure 2). Notably, this phenomenon was observed in the platform incorporating THP1-M0 macrophages (Figure 2).

### 3.3. Endothelium Barrier Function and Permeability

**Figure 3:**
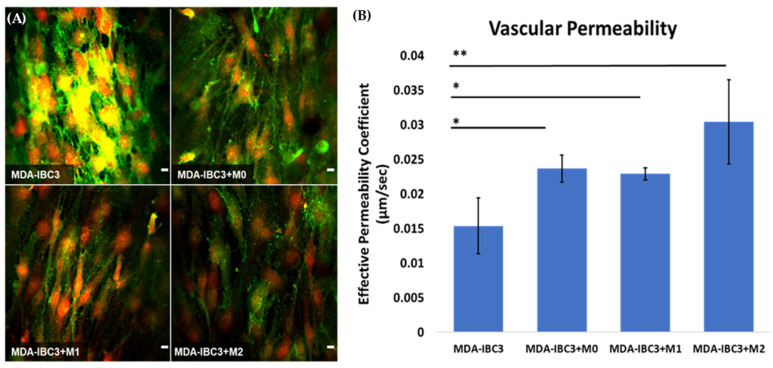
Assessing the impact of macrophage-tumor-vessel interactions on endothelial barrier function using two measurements: (**A**) visualization of the endothelial barrier with PECAM-1 (green) staining with DAPI (red) staining of cell nuclei for analyzing cell–cell junction between neighboring TIME cells, (**B**) quantification of the effective permeability of the endothelial barrier based on dextran permeability measurements (* *p* < 0.05, ** *p* < 0.01). Scale bar: 10 µm.

To assess the impact of macrophage-tumor-vessel interactions on endothelial barrier function, we quantified Platelet Endothelial Cell Adhesion Molecule 1 (PECAM-1) staining and conducted dextran permeability measurements on the vascular vessel within each platform. These analyses were performed to evaluate the influence of these interactions on the integrity of the endothelial barrier, as demonstrated in Figure 3. The presence of THP-1 macrophages in the platform resulted in a significantly more permeable endothelium compared to the control MDA-IBC3 platform without macrophages, as indicated by PECAM downregulation, potentially highlighting a functional role as well. The permeability of platforms containing M0 and M1 THP-1 macrophages was found to be 1.5 times higher (*p* < 0.05), while platforms with M2 THP-1 macrophages showed a 2-fold increase in permeability (*p* < 0.01) compared to MDA-IBC3 platforms without THP-1 macrophages, highlighting the significance of TAMs in promoting vascular permeability potentially to the extent needed to permit emboli to intravasate.

### 3.4. Collagen ECM Porosity

**Figure 4:**
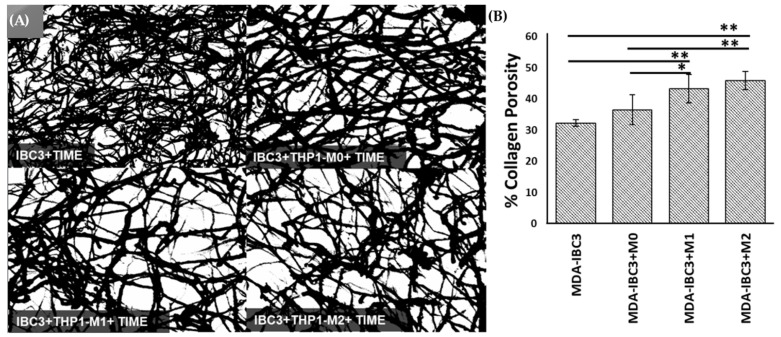
Scanning Electron Microscopy (SEM) images showing the collagen matrix (as a function of culturing macrophages of varying polarization state (**A**). The porosity of the collagen matrix was determined by calculating measurements from SEM images of the extracellular matrix (ECM) (**B**), * *p* < 0.05, ** *p* < 0.01.

Collagen in tissues presents a physical barrier to tumor cell migration and invasion [49]. However, collagen degradation can lead to an increase in porosity, permitting collective tumor cell migration and intravasation. Collective cancer cell migration refers to the coordinated movement of groups of cancer cells as they invade surrounding tissues and spread to distant sites. In IBC, collective cancer cell migration is particularly relevant due to the unique clinical presentation of the disease [50,51,52]. The analysis of porosity based on scanning electron microscopy (SEM) images in Figure 4 demonstrated the impact of THP1 macrophages on matrix remodeling. Platforms containing THP-1 M1 or M2 macrophages exhibited significantly higher porosity compared to platforms with THP-1 M0 macrophages and those consisting of MDA-IBC3 cells alone. M2-conditioned macrophages in the MDA-IBC3+M2 platform led to the highest increase in matrix porosity over MDA-IBC3 alone, with a 1.5-fold increase (*p* < 0.01), followed by the M1 macrophages in the MDA-IBC3+M1 platform with a 1.3-fold increase over MDA-IBC3 (*p* < 0.01). While the matrix porosity in the MDA-IBC3+M0 platform had a higher mean porosity compared to MDA-IBC3, it was not found to be significant, highlighting the importance of macrophage phenotype in matrix remodeling.

### 3.5. Cytokine Analyses

**Figure 5:**
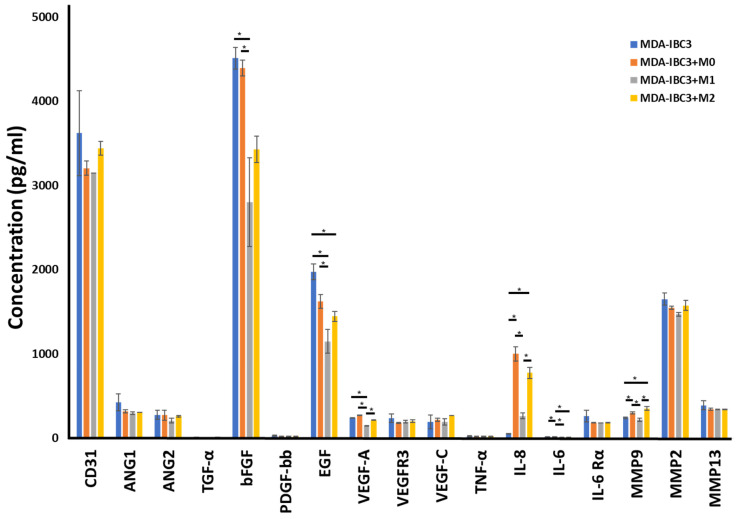
Cytokine measurements on the circulating media collected from perfusion outflow after 78 h of graded flow protocol in MDA-IBC3, MDA-IBC3+M0, MDA-IBC3+M1, and MDA-IBC3+M2 using Luminex assay, * *p* < 0.05.

To survey possible mediators of the macrophage effects on the stroma, we performed cytokine analysis on the circulating media collected from perfusion outflow after 78 h of graded flow protocol (Figure 5). bFGF and EGF were both broadly reduced in macrophage co-culture across macrophage phenotypes. CD31, ANG1, and ANG2 were not significantly changed in macrophage co-cultured platforms. IL-8 is strongly increased across macrophage co-cultured, especially in M2 and M0. MMP9 also demonstrates a selective increase in the M0 and M2 co-cultured platforms, while MMP2 and MMP13 are not significantly changed.

### 3.6. Macrophage Induced Endothelial Sprouting

To test the influence of macrophages on endothelial sprouting, THP1-derived M0 macrophages were cultured with TIME cells alone or in SUM149 platforms, where we have previously reported no observable endothelial sprouting in the presence of the IBC tumor cells alone [37]. Co-culture with M0 macrophages resulted in sprouting of the endothelium in the absence of IBC cells, to a lesser extent than was observed in co-culture in platforms with MDA-IBC3 cells. Whereas the inclusion of M0 macrophages in the MDA- IBC3 platforms results in a synergistic increase in angiogenic sprouting, the comparable inclusion of M0 cells in the SUM149 platforms demonstrates angiogenic sprouting attributable to M0 alone. Nevertheless, on cytokine analysis, MDA-IBC3 and, to a lesser extent, SUM149 both increase the secretion of IL-8 and MMP-9 over M0 alone, demonstrating an increased modulation of these cytokines.

**Figure 6:**
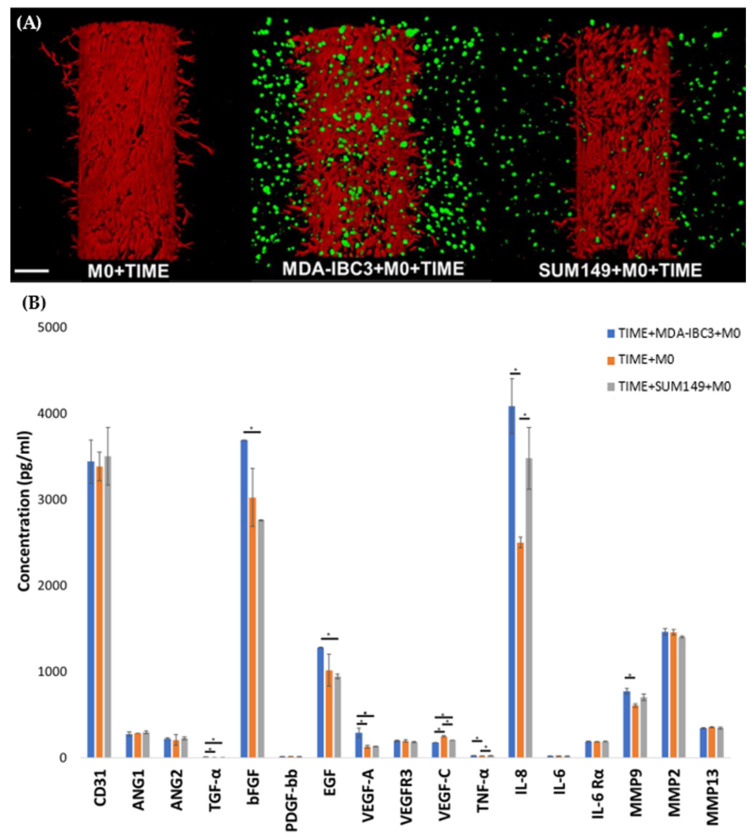
(**A**) Confocal images of in vitro vascularized breast tumor platforms consisting of MDA-IBC3 cells, TIME cells, and SUM 149 cells, cultured with M0 macrophages. Cancer cells are depicted in green, while TIME cells are shown in red; (**B**) Cytokine measurements on the circulating media collected from perfusion outflow after 78 h of graded flow protocol in the TIME+MDA-IBC3+M0, TIME+M0, and TIME+SUM149+M0 and platforms using Luminex assay, * *p* < 0.05.

Cytokine analysis was performed to identify cytokines altered by combining tumor cells and macrophages compared to macrophages alone. TNG-a, IL-8, and MMP9 are significantly increased in both MDA-IBC3 and SUM149 platforms over macrophage culture alone (Figure 6).

## 4. Discussion

Macrophages have been implicated in the phenotype and progression of IBC in many studies [22,25,53,54]. Here, we examined the effect of macrophages on vascular development and matrix structure using a 3D microfluidic platform and found that macrophages, particularly IL4 and IL13 stimulated macrophages, enhance sprouting observed from endothelial cells cultured with IBC cells, enhance collagen porosity, and promote tumor emboli intravasation into the vessel. This finding is consistent with previous studies demonstrating that macrophages promote angiogenesis and extracellular matrix remodeling in various types of cancer [20,55]. Cytokines preferentially secreted in M0 and M2 co-culture platforms were IL8 and MMP-9 which is consistent with the known role of these cytokines in promoting angiogenesis and tumor invasion [56,57]. While both macrophages and IBC cells can promote TIME sprouting independently, the combination of macrophages and IBC cells demonstrates enhanced secretion of IL8 and MMP9, which are known to promote angiogenesis and tumor invasion.

In line with our findings, a study conducted by Lin et al. using the PyMT mouse model demonstrated a notable increase in the formation of new sprouts as a result of THP-1 macrophages’ presence. Furthermore, this study revealed that macrophages play a crucial role in regulating the development of high-density vascular networks and the progression from benign to invasive tumors [58,59]. Previous research has indicated a correlation between a high population of tumor-associated macrophages (TAMs) and an elevated mean vessel density in individuals diagnosed with invasive breast cancer [60,61]. This study demonstrated that the inclusion of macrophages, regardless of their specific phenotype, led to a significant elevation in vessel density compared to the control group. Additionally, the presence of macrophages induced the sprouting of vessels in platforms. While previous studies have primarily linked angiogenesis to the M2 phenotype, it is worth noting that the M1 phenotype has also been found to possess angiogenic capabilities and express proangiogenic growth factors [62,63], correlating with results from our platforms. Moreover, recent research has embraced the notion that tumor-associated macrophages (TAMs) express markers associated with both M1 and M2 phenotypes [64,65].

We also noted a significant impact of macrophages on vessel permeability. The presence of THP1-derived macrophages led to a decrease in the expression of intercellular junction protein PECAM-1 in the endothelial cells, which subsequently resulted in increased vascular permeability. These observations provide evidence suggesting that macrophages may exert an immunosuppressive effect on endothelial cells during the process of leukocyte transendothelial migration [66,67]. A study demonstrated that the presence of macrophages resulted in an increase in endothelial permeability. They observed that macrophages released cytokines that led to the disorganization and degradation of junction proteins in the endothelial cells, contributing to the observed changes in permeability [68]. Another study employed intravital high-resolution two-photon microscopy to demonstrate the hyperpermeability of the vasculature at sites where tumor cells, macrophages, and blood vessels interacted [69,70]. Their findings indicated that the increased permeability facilitated tumor metastasis, similar to the behavior observed in our THP1-M0 platforms. In the MDA-IBC3+M0 platform, we observed indications of tumor intravasation, which was not observed in the MDA-IBC3 only platforms. MDA-IBC3 cells were observed as a cluster on the luminal side of the endothelial vessel, indicating their intravasation across the endothelium. Furthermore, other in vivo imaging studies employing multiphoton microscopy have revealed instances of intravasation occurring in tumor cells associated with macrophages located near blood vessels [16,47,71]; earlier investigations in IBC have revealed that the media conditioned by macrophages can trigger a migratory and invasive phenotype in IBC cells [6,9]. Furthermore, we did not consistently observe this intravasation behavior in our platforms, which aligns with previous studies indicating that transient changes in vessel permeability induced by macrophage interactions are associated with intravasation.

Cytokine analysis of the interactions between MDA-IBC3, THP-1, and TIME cells revealed an enhanced expression of angiogenic factors in the presence of THP1 macrophages. Surprisingly, platforms with THP-1 M1 macrophages exhibited a notable reduction in the expression of VEGF and bFGF, which are typically known to promote angiogenesis. However, despite this decrease, these platforms displayed an increased occurrence of angiogenic sprouting compared to MDA-IBC3 platforms. Notably, IL-8, which was upregulated in the M1 platforms, has been demonstrated in previous studies to stimulate tumor angiogenesis, metastasis, and proliferation [72]. Previous research has demonstrated that the expression of IL-8 can stimulate endothelial cell proliferation and the formation of capillary tubes, which aligns with our observations in the platforms where THP1 cells exhibited increased IL-8 expression [48,56,72,73,74]. IL-6 and MMP9 were among the factors that exhibited significant upregulation. It is worth noting that macrophages are known to be a strong source of MMP9, which has been linked to the enhanced degradation of the extracellular matrix [74,75,76,77]. The observed increase in MMP9 expression in the THP1 platforms was consistent with the higher collagen porosity observed, indicating the matrix-degrading properties of MMP9. Previous studies have established a connection between increasing scaffold pore size and improved vascularization [78,79,80]. In addition, MMP9 has also been involved in activating membrane-bound growth factors. When comparing the impact of THP1 M0 macrophages on the MDA-IBC3, TIME, and SUM149 platforms, we observed an increase in various angiogenic factors, including TGF-α, bFGF, EGF, VEGF-A, IL-8, and MMP9, specifically in the MDA-IBC3 platforms. This increase in angiogenic factors correlated with the observed augmentation in angiogenic sprouting. Previous studies have also reported an upregulation of IL-8 in HER2+ breast cancers, which aligns with our findings, where the expression of IL-8 in the HER2+ MDA-IBC3+M0 platform was notably higher compared to the triple-negative SUM149+M0 platforms [48,72,81]. Furthermore, MMP9 levels were elevated in both IBC platforms, MDA-IBC3+M0 and SUM149+M0, in comparison to the TIME+M0 platform.

In summary, our study aimed to improve the complexity of our existing in vitro IBC model by incorporating macrophages, thus emulating relevant physiological features observed in patients. The inclusion of macrophages, regardless of their specific phenotype, had a significant impact on both vessel sprouting and matrix remodeling, effectively capturing characteristic behaviors associated with IBC, such as the intravasation of IBC cells in clusters. This validates existing in vivo data by demonstrating that the introduction of macrophages enhances the Inflammatory Breast Cancer (IBC) phenotype. This observation correlates with our prior published data, where in vitro investigations involving the murine macrophage cell line RAW 264.7 were substantiated in vivo. This validation reinforces the impact of blocking physiologic macrophages in mediating IBC-like skin invasion and local recurrence [25]. Notably, we observed a significant divergence in the angiogenic response to macrophages. However, the precise underlying reasons for this discrepancy remain speculative, warranting further analysis and investigation to elucidate the mechanisms involved in this phenomenon.

## 5. Conclusions

In this study, we expanded upon our previously established 3D in vitro microfluidic platform incorporating THP1 M0, M1, or M2 macrophages and reproducing phenotypes observed in vivo. The involvement of macrophages in IBC progression has been established, and our developed platform allowed us to demonstrate several significant findings. Specifically, we observed an increase in angiogenic sprouting, vessel permeability, matrix porosity, and the expression of tumorigenic factors, all of which contribute to tumor growth and eventual metastasis. Additionally, the presence of THP1 M0 macrophages in our platform led to the intravasation of IBC cells, validating the behaviors observed in previous in vivo and in vitro studies. This in vitro platform offers a straightforward and effective means to investigate and assess the key factors contributing to the aggressive nature of IBC, ultimately aiding in the development of targeted treatments specifically for IBC.

## Data Availability

Not applicable.
